# Supplementary material for: PRMT5 inhibition disrupts splicing and stemness in glioblastoma
Source: Nat Commun. 2021 Feb 12;12:979. doi: 10.1038/s41467-021-21204-5 (PMC7881162; doi:10.1038/s41467-021-21204-5)
Supplement: Supplementary file 1 — Supplementary Information [file 41467_2021_21204_MOESM1_ESM.pdf]

PRMT5 inhibition disrupts splicing and stemness in glioblastoma

Sachamitr et al.

Supplementary Information:

Supplementary figure 1

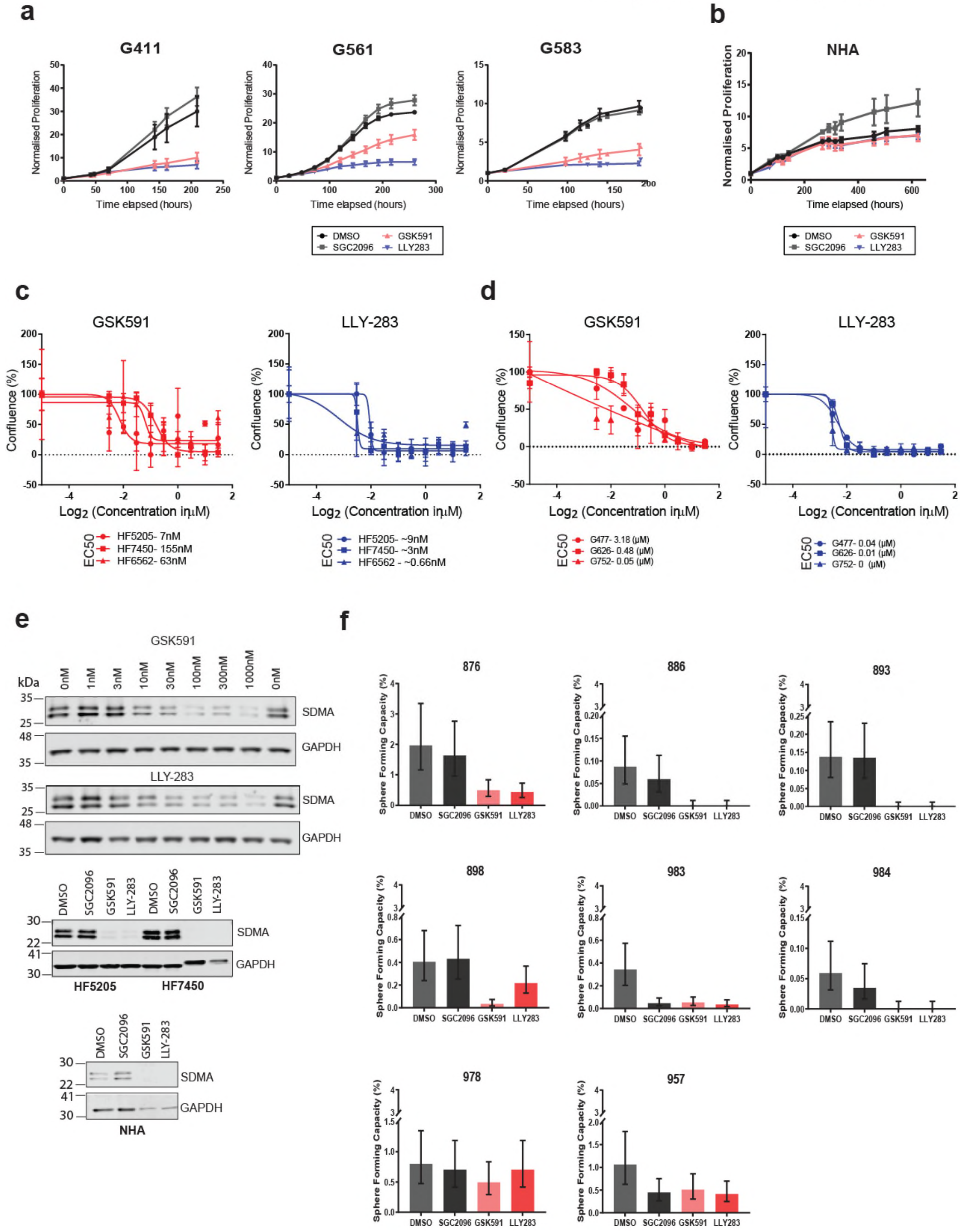

Supplementary Figure 1: PRMT5 inhibitors impair GSC and HFS cell proliferation and sphere forming capacity of primary GBM samples. **a** Relative cell confluence of three GSC lines, G561, G583 and G411, treated with PRMT5 inhibitors, 1 μM GSK591 and 1 μM LLY-283, and controls, 1 μM SGC2096 and 0.05% DMSO. Cell confluence values were normalised to the confluency at the first time point. Mean ± SD. N=3 technical replicates; data shown are representative of 3 independent experiments. **b** Relative cell proliferation of normal human astrocyte (NHA) cells, treated with 1 μM of the PRMT5 inhibitors, GSK591 and LLY-283, and controls, SGC2096 and DMSO. Cell confluence values were normalised to the confluency at the first time point. N=3 technical replicates; data shown are representative of 3 independent experiments, Mean ± SD. **c** Dose-response of three HFNS lines upon treatment with GSK591 (red) and LLY-283 (blue), with doses ranging from 3 nM to 30 μM. Data shown are representative of 3 independent experiments, mean ± SD. **d** Dose-response curves of three pediatric GSC lines upon treatment with GSK591 (red) and LLY-283 (blue), with doses ranging from 3 nM to 30 μM. Data shown are representative of 3 independent experiments, mean ± SD. **e** Representative western blots of the SDMA mark on the SmB/B' protein of GSC line G583 following 5-day treatment with the indicated doses of GSK591 and LLY-283 (top panel) and of two human fetal neural stem cell (HFNS) lines and normal human astrocytes (NHAs) treated with 1 μM of the PRMT5 inhibitors, GSK591 or LLY-283, and controls, SGC2096 and DMSO (bottom panel). The Westerns were repeated two independent times with similar results. **f** Limiting dilution analysis (LDA) performed on freshly dissociated GBM cells from nine patients. Cells were treated with 1 μM of the PRMT5 inhibitors, GSK591 and LLY-283, and controls, SGC2096 and DMSO, for 21 days. Data shows percentage sphere-forming capacity, N=1. The bar graphs represent the mean sphere forming frequencies and the error bars represent the upper and lower limits. Source data are provided as a Source Data file.

Supplementary figure 2

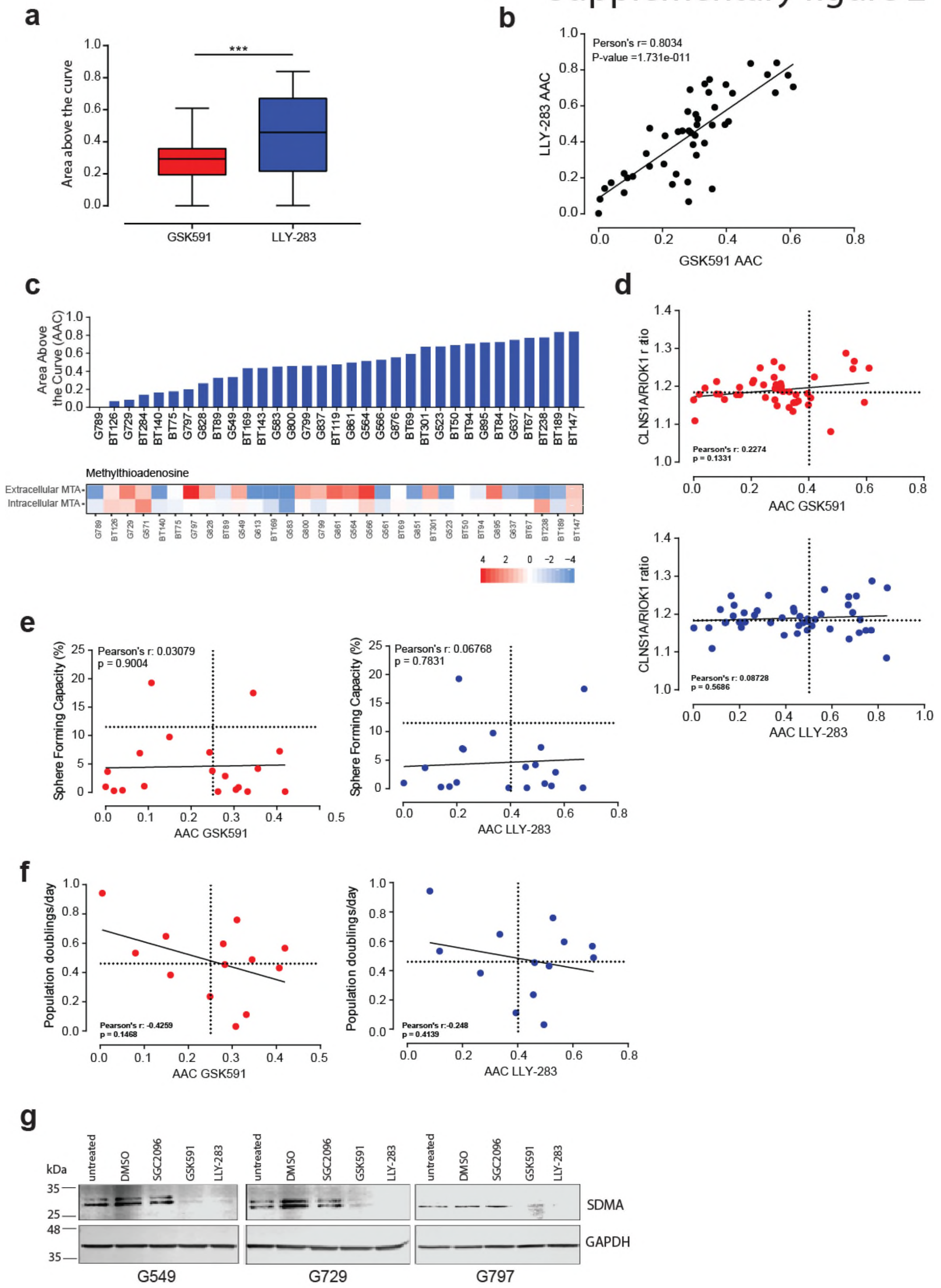

Supplementary Figure 2: Pharmacogenomic and biomarker analysis of PRMT5 inhibitors. **a** Drug sensitivity comparison between GSK591 and LLY-283. Unpaired t test with Welch's correction- two tailed, P value=0.0004. N=46 biologically independent GBM patient-derived samples. **b** Correlation of response (based on AAC) to GSK591 and LLY-283 across all GSC lines. Pearson r=0.8034, 95% confidence interval=0.6691 to 0.8869, R squared=0.6454; P value (two-tailed)=1.731e-011, significant (alpha = 0.05); number of XY Pairs=46. **c** Bar graph of the AAC values for LLY-283 in a panel of 33 GSCs (top panel). Heatmap representing the intracellular and extracellular methylthioadenosine (MTA) concentrations derived from metabolomics analysis from the same panel of GSC lines. The data represent the mean of 6 biological replicates per sample. **d** Correlation between Area Above the Curve (AAC) for the PRMT5 inhibitors (GSK591: red; LLY-283: blue) and CLNS1A/RIOK1 ratio for 45 GSC and BT lines. **e** Correlation between AAC for the PRMT5 inhibitors (GSK591: red; LLY-283: blue) and sphere-forming capacity for 20 GSC lines. **f** Correlation between AAC for the PRMT5 inhibitors (GSK591: red; LLY-283: blue) and population doublings/day for 13 GSC lines. **g** Western blots of the SDMA mark in cellular extracts from a panel of 3 poor responder GSCs G729, G797 and G549 following 1 μM GSK591, LLY-283 or control treatments for 7 days. The Westerns were repeated three independent times with similar results. Source data are provided as a Source Data file.

Supplementary figure 3

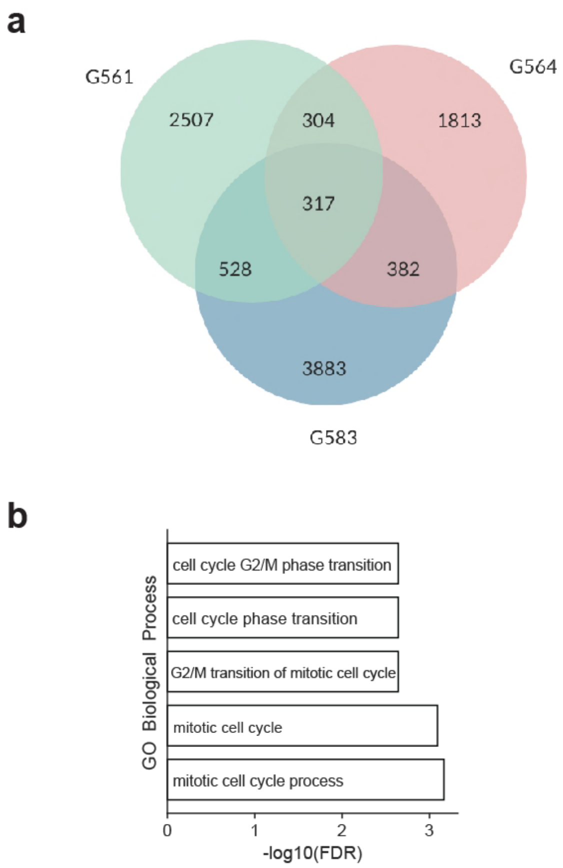

Supplementary Figure 3: Global effect of PRMT5 probes on alternative splicing and cell cycle. a Venn diagram depicting the alternative splicing events (ASEs) identified after GSK591 treatment in the three GSC lines. **b** Enrichment analysis for transcripts containing disruptive alternative splicing events for Gene Ontology (GO) Biological Process. The y-axis shows the top 5 enriched classes for each type, ranked by adjusted p value (by False Discovery Rate, FDR) from low to high. The x-axis shows the FDR value transformed in a -log10 scale for visualization purposes. Source data are provided as a Source Data file.

Supplementary Figure 4

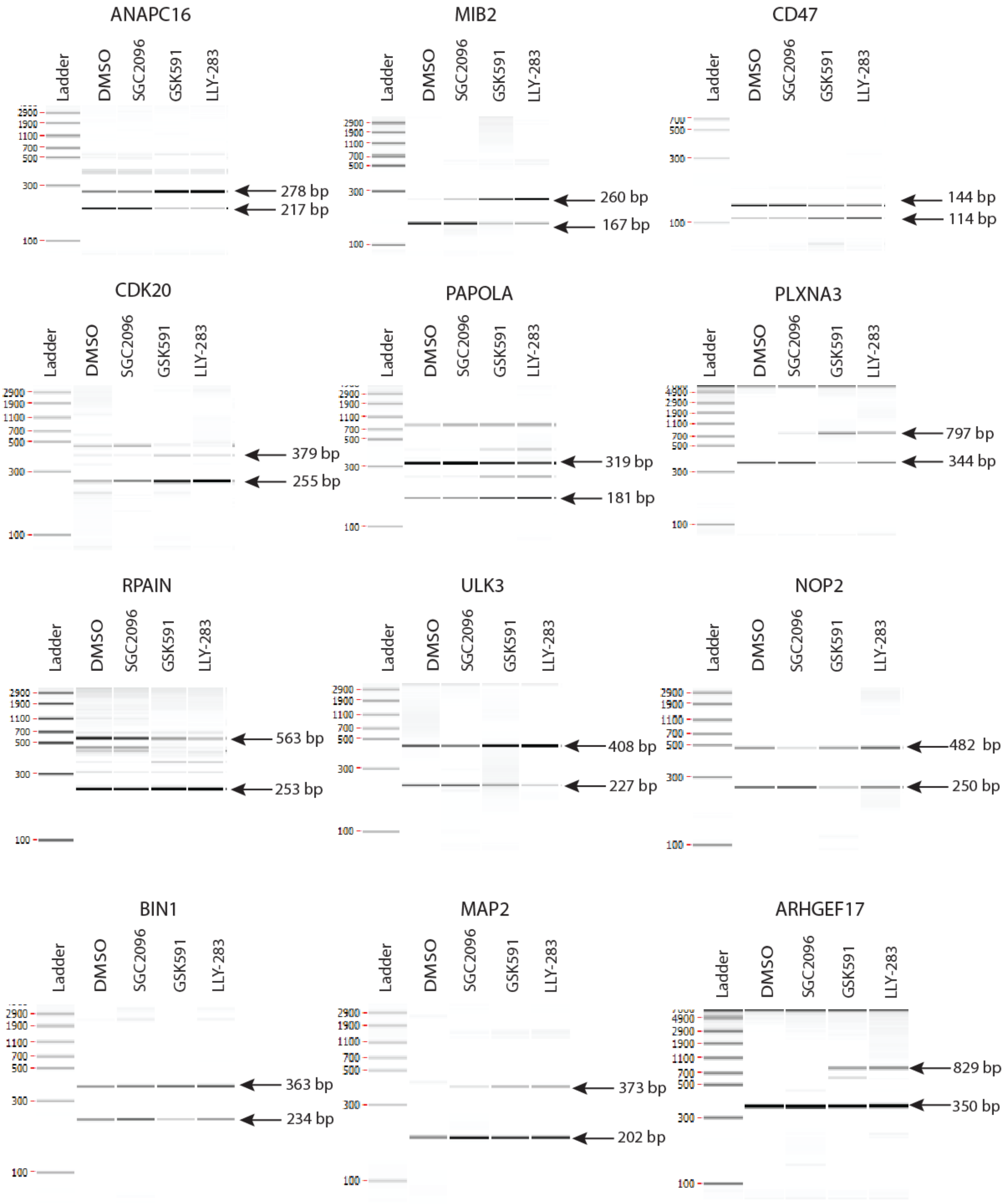

Supplementary Figure 4. **RT-PCR validation of select PRMT5i-induced splicing changes.** RT-PCRs for the indicated gene isoforms were performed using total RNA from the G561 patient-derived cell line following 72 hrs treatment with the indicated PRMT5 probes or controls. The amplified products were resolved using capillary electrophoresis alongside DNA ladder standards. The sizes of the two expected amplicons corresponding to the long and short isoforms for each gene are indicated. The molarity of each band was calculated using Caliper software and the corresponding PSI values were calculated. The details for each ASE including the primer sequences used to amplify them are indicated in Table ST4. The RT-PCR experiments were repeated independently three times with similar results. Source data are provided as a Source Data file.

Supplementary figure 5

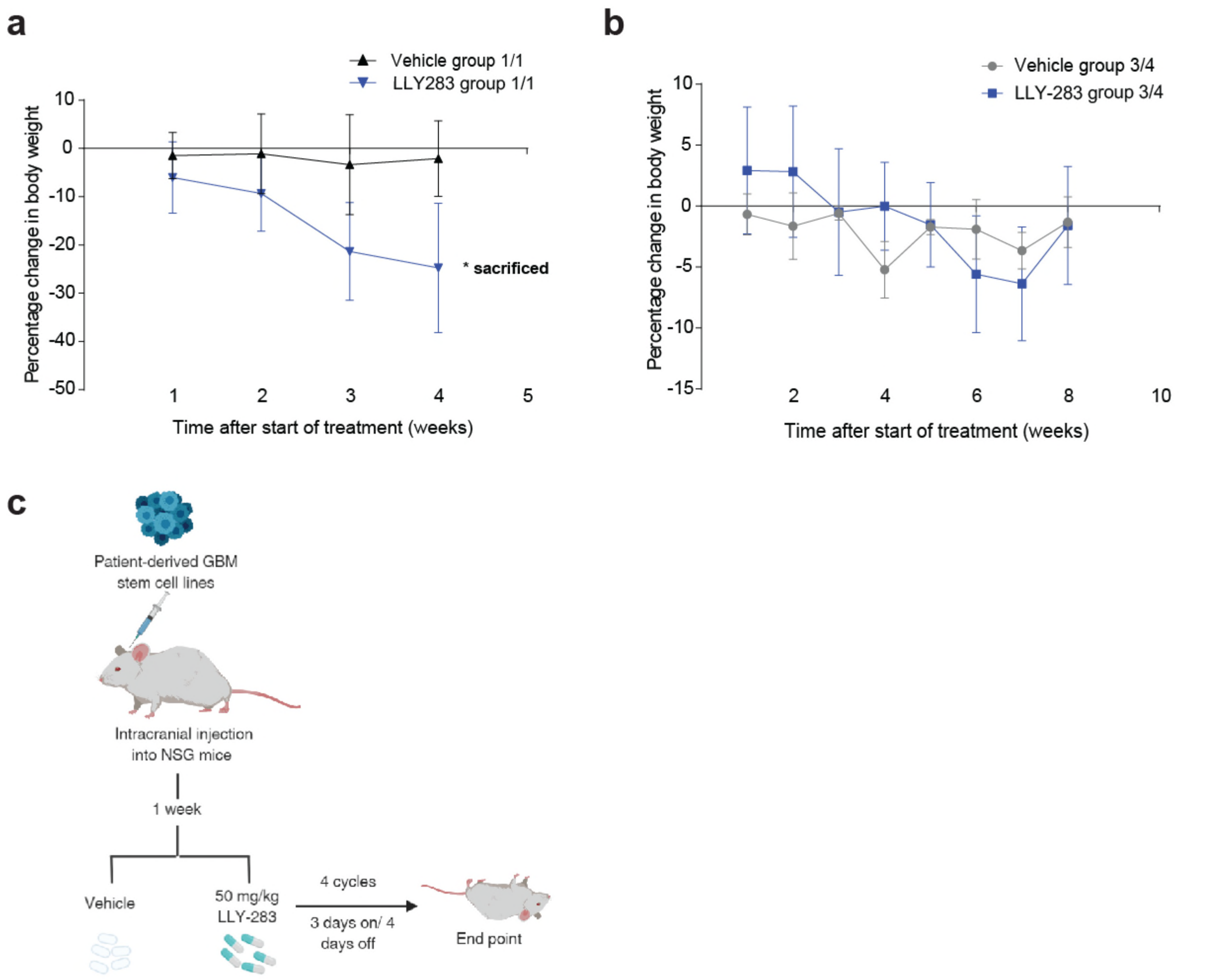

Supplementary Figure 5: LLY-283 dosing and pharmacotoxicity studies. (a) Percentage change in body weight of NSG mice dosed with 50 mg/kg LLY-283 (administered every other day) over 8 weeks. n = 3, mean ± SD. **(b)** Percentage change in body weight of NSG mice dosed with 50 mg/kg LLY-283 (administered 3 days on and 4 days off) over 8 weeks. n = 3, mean ± SD. **(c)** Schematic showing workflow of orthotopic *in vivo* assay investigating the effect of LLY-283 on the survival of NSG mice. The mouse image was created using Biorender. Source data are provided as a Source Data file.

Supplementary table 1

Protein Family	Specific target	Probe	PubMed ID
2OG	PHD2	IOX2	23683440
Arginine deiminases	PADI4	GSK484	25622091
Bromodomain	BRD9/7	LP99	25864491
Bromodomain	BET family	(+)-JQ1	20871596
Bromodomain	BAZ2A/2B	BAZ2-ICR	25719566
Bromodomain	BRD9/7	BI-9564	26914985
Bromodomain	BAZ2A/2B	GSK2801	25799074
Bromodomain	BRD: BRD9	I-BRD9	25856009
Bromodomain	CREBBP/EP301	I-CBP112	26552700
Bromodomain	BPRF1/2/4	NI-57	
Bromodomain	CECR2	NVS-CECR2-1	
Bromodomain	BPRF1/2/3	OF-1	
Bromodomain	SMARCA2/4	PFI-3	26139243
Bromodomain	BRPF1B	PFI-4	
Bromodomain	CREBBP/EP300	SGC-CBP30	24946055
Bromodomain	D2, BRD3, BRD4, BRDT (B	PFI-1	23576556
Dehydrogenase	IDH1 mutant	GSK864	26436839
Histone Deacetylase	HDAC 1/ 2/ 3/8	CI-994	
Lysine Demethylase	pan-2-OG	IOX1	24504543
Lysine Demethylase	KDM: JMJD3/UTX	GSK-J4*/J1	22842901
Lysine Demethylase	KDM: LSD1	GSK-LSD1	26175415
Methyl Lysine Binder	EED	A-395	28135237
Methyl Lysine Binder	L3MBTL3	UNC1215	23292653
Methyltransferase	SUV420H1/H2	A-196	28114273
Methyltransferase	G9a/GLP	A-366	24900801
Methyltransferase	SMYD2	BAY-598	27075367
Methyltransferase	EZH2	GSK343	24900432
Methyltransferase	PRMT5	GSK591	26985292
Methyltransferase	PRMT5	LLY-283	30034588
Methyltransferase	PRMT type 1	MS023	26598975
Methyltransferase	PRMT4/6	MS049	27584694
Methyltransferase	SETD7	PFI-2	25136132
Methyltransferase	DOT1L	SGC0946	23250418
Methyltransferase	PRMT3	SGC707	25728001
Methyltransferase	G9a, GLP	UNC0638	21743462
Methyltransferase	G9a/GLP	UNC0642	24102134
Methyltransferase	EZH2/H1	UNC1999	23614352
Poly (ADP-ribose) polymerase	PARP1/2/3	Olaparib	18800822
WD40	WDR5	OICR-9429	26167872

Supplementary Table 1: List of small molecule inhibitors in the epigenetic probe library used for screening GSCs, alongside their protein family, specific target, and publication details.

	GSK591		LLY283	
GNS Line	IC50 (µM)	AAC	IC50 (µM)	AAC
G789	NA	0.00	55.22	0.00
BT126	2125.07	0.28	NA	0.07
G729	45.57	0.00	NA	0.08
G571	NA	0.08	NA	0.12
BT284	591.41	0.36	NA	0.14
G706	39.00	0.02	NA	0.14
BT140	NA	0.23	NA	0.16
G594	NA	0.04	NA	0.17
BT75	NA	0.28	NA	0.18
G797	NA	0.09	NA	0.20
G489	NA	0.11	NA	0.21
G584	NA	0.24	NA	0.22
G567	29.33	0.08	35066257.38	0.22
G828	13.07	0.16	NA	0.26
G361	NA	0.20	NA	0.28
BT89	1499.73	0.31	NA	0.33
G549	144.95	0.15	NA	0.33
G719	9.23	0.30	NA	0.38
G613	19.20	0.33	NA	0.39
BT169	NA	0.21	NA	0.43
BT143	0.60	0.30	0.16	0.44
G583	1.34	0.29	0.12	0.45
G800	16.40	0.25	0.12	0.46
G799	0.68	0.28	0.02	0.46
G837	5.01	0.26	0.04	0.46
BT119	8.64	0.16	0.23	0.48
G411	1.50	0.36	0.04	0.49
G702	1.62	0.31	0.02	0.49
G861	1.28	0.40	0.04	0.50
G564	0.23	0.41	0.03	0.51
G566	2.05	0.31	0.03	0.53
G876	2.45	0.30	0.03	0.55
G561	2.77	0.28	0.01	0.57
BT69	0.69	0.36	0.03	0.59
G851	0.57	0.42	0.01	0.67
BT301	0.10	0.55	0.00	0.67
G523	0.83	0.35	0.01	0.67
BT50	NA	0.29	0.02	0.69
BT94	0.06	0.61	0.00	0.71
G895	0.64	0.40	0.01	0.72
BT84	NA	0.33	0.01	0.72
G637	0.34	0.35	0.00	0.75
BT67	0.05	0.59	0.00	0.77
BT238	0.15	0.53	0.00	0.77
BT189	0.11	0.48	0.00	0.84
BT147	0.17	0.56	0.01	0.84

Supplementary Table 2: EC50s and AACs calculated from dose-response assays across 46 patient-derived GSC lines for GSK591 and LLY-283 over a range of compound concentrations from 3 nM to 30 µM. Higher AAC represents greater sensitivity.

Gene	Primer Name		Primer Sequence	
	Forward	Reverse	Forward	Reverse
ANAPC16	ANAPC16.e.pp.F	ANAPC16.e.pp.R	GGCTCCGTCTGTTGGGGG	CATCTCTCCAGCTCCTTTGGG
ANAPC16	ANAPC16.e.pp.F	ANAPC16.e2.pp.R	GGCTCCGTCTGTTGGGGG	TGTTTCACCTGTTTAAGCGTGGA
ARHGEF17	ARHGEF17.e.pp.F	ARHGEF17.e.pp.R	GGTGCCCTTTGACAGTGACTC	AGCCATCCTCAGTGCCCAG
BIN1	BIN1.e.pp.F	BIN1.e.pp.R	GACAACGCGCCTGCAAAAG	GGAGGCTGCTTCACTTGCC
CD47	CD47.e.pp.F	CD47.e.pp.R	CAATGCATGGCCCTCTTCTGA	CGGAGTCCATCACTTCACTTCA
CDK20	CDK20.e.pp.F	CDK20.e.pp.R	AAACCTGCCAACCTGCTCATC	GCTGGTGAGGAGGGTAGAGAA
LUC7L	LUC7L.e.pp.F	LUC7L.e.pp.R	CCTGCCTGAGAGAAGTCGTCG	GTGACTCTTGCAGACACGGTC
MAP2	MAP2.e.pp.F	MAP2.e.pp.R	CAGCAGGTGGGGAATCAGCT	CGCCGTGCTGAAGAAGAGATA
MIB2	MIB2.e.pp.F	MIB2.e.pp.R	ATTGTCGAGGTCCTCACGGAG	GTGAAGCCGTCCTCCTTCTTG
MKI67	MKI67.e.pp.F	MKI67.e.pp.R	GAGCCAGCACGTCGTGTC	CTTGATGATTTTCTTCAGGACAGGTG
NOP2	NOP2.e.pp.F	NOP2.e.pp.R	GACTCTGTCAATGCGACCTCC	TGGCAATGAAGAACCCATCCA
PAPOLA	PAPOLA.e.pp.F	PAPOLA.e.pp.R	ACCCACCACCTAGATCTTCAGG	GCAGGGAGAGCAGGGATATCA
PLXNA3	PLXNA3.e.pp.F	PLXNA3.e.pp.R	TCACCATCTTCTCTCAGGGCC	CGTGCCAATGAAGACCACAGA
RPAIN	RPAIN.e.pp.F	RPAIN.e1.pp.R	CATGGCTGTGCTGGAGGAAAT	ACCTGCGTCACAAAAGACCAA
RPAIN	RPAIN.e.pp.F	RPAIN.e2.pp.R	CATGGCTGTGCTGGAGGAAAT	GGCTCCTCAGAGGGAATGAGT
ULK3	ULK3.e.pp.F	ULK3.e.pp.R	ACTTTGGTTTCGCACAACACA	CTCGATGACCCGGTTGCTAC

Supplementary Table 3. Primer sequences used for RT-PCR.
