# Supplementary material for: PRMT5 inhibition disrupts splicing and stemness in glioblastoma
Source: Nat Commun. 2021 Feb 12;12:979. doi: 10.1038/s41467-021-21204-5 (PMC7881162; doi:10.1038/s41467-021-21204-5)
Supplement: Supplementary file 3 — Description of Additional Supplementary Files [file 41467_2021_21204_MOESM3_ESM.pdf]

Description of Additional Supplementary Files

Supplementary Data 1: PRMT5i-dependent ASEs annotation. Detailed genomic coordinates and PSI values for each ASE type across GSC lines are provided in separate tabs. For a summary of all ASEs across all 3 GSCs, see first tab. The predictive ASE signature information and genomic coordinates is located in the tabs labelled ‘Drug response ASEs’.

Supplementary Data 2: Fold change after PRMT5 inhibition for RNA sequencing and proteomics. GSC lines G561, G564 and G583 treated with inactive control SGC2096 or PRMT5 inhibitors GSK591 (RNA-Seq and proteomics) or LLY283 (proteomics) are considered. ASE refers to genes displaying significant alternative splicing events.

Supplementary Data 3: Fold-enrichment of genes displaying ASE among the most downregulated proteins after PRMT5 inhibition.

Supplementary Data 4: **Detailed PSI values per GSC line sample for the RT-PCR validated ASEs.**
