# Supplementary material for: PRMT5 inhibition disrupts splicing and stemness in glioblastoma
Source: Nat Commun. 2021 Feb 12;12:979. doi: 10.1038/s41467-021-21204-5 (PMC7881162; doi:10.1038/s41467-021-21204-5)
Supplement: Supplementary file 8 — Reporting Summary [file 41467_2021_21204_MOESM8_ESM.pdf]

nature research

Corresponding author(s): Panagiotis Prinos, Cheryl Arrowsmith, Peter Dirks,

Last updated by author(s): Nov 20, 2020

Reporting Summary

Nature Research wishes to improve the reproducibility of the work that we publish. This form provides structure for consistency and transparency in reporting. For further information on Nature Research policies, see Authors & Referees and the Editorial Policy Checklist .

Statistics

For all statistical analyses, confirm that the following items are present in the figure legend, table legend, main text, or Methods section.

n/a	Confirmed	
☐	☒	The exact sample size (*n*) for each experimental group/condition, given as a discrete number and unit of measurement
☐	☒	A statement on whether measurements were taken from distinct samples or whether the same sample was measured repeatedly
☐	☒	The statistical test(s) used AND whether they are one- or two-sided<br>*Only common tests should be described solely by name; describe more complex techniques in the Methods section.*
☒	☐	A description of all covariates tested
☒	☐	A description of any assumptions or corrections, such as tests of normality and adjustment for multiple comparisons
☐	☒	A full description of the statistical parameters including central tendency (e.g. means) or other basic estimates (e.g. regression coefficient) AND variation (e.g. standard deviation) or associated estimates of uncertainty (e.g. confidence intervals)
☐	☒	For null hypothesis testing, the test statistic (e.g. *F*, *t*, *r*) with confidence intervals, effect sizes, degrees of freedom and *P* value noted<br>*Give P values as exact values whenever suitable.*
☒	☐	For Bayesian analysis, information on the choice of priors and Markov chain Monte Carlo settings
☒	☐	For hierarchical and complex designs, identification of the appropriate level for tests and full reporting of outcomes
☐	☒	Estimates of effect sizes (e.g. Cohen's *d*, Pearson's *r*), indicating how they were calculated

Our web collection on statistics for biologists contains articles on many of the points above.

Software and code

Policy information about availability of computer code

Data collection	Incucyte Zoom software v2016B and v2018A, IncucyteDRC package
Data analysis	Graph Pad Prism 7, DESeq2 package v3.8.6, GSEA, Cytoscape v3.6.1, GSVA, FastQC v0.11.8, STAR aligner v2.6.1 , samtools flagstat v1.8, Stringtie v.1.3.4, MATS v3.2.5, IRFinder v.1.2.4, STRINGdb v.10.5, VastDB (hg38 assembly), CPAT v.1.2.4, PharmacoGx package, PEAKS, Extreme Limiting Dilution Assay (ELDA), Caliper LabChip GX version 5.2.2009, R software v3.4.4

For manuscripts utilizing custom algorithms or software that are central to the research but not yet described in published literature, software must be made available to editors/reviewers. We strongly encourage code deposition in a community repository (e.g. GitHub). See the Nature Research guidelines for submitting code & software for further information.

The code used for calculating the EC50s from Incucyte confluence data is available at https://github.com/bhklab/PRMT5i_GBM.

Data

Policy information about availability of data

All manuscripts must include a data availability statement. This statement should provide the following information, where applicable:

- Accession codes, unique identifiers, or web links for publicly available datasets
- A list of figures that have associated raw data
- A description of any restrictions on data availability

RNA-Seq and WGS data for GSC lines reported in this manuscript have been deposited at the European Genome-phenome Archive, EGA study ID EGAS00001004395 [https://www.ebi.ac.uk/ega/search/site/EGAS00001004395].The RNA-seq raw data files for the three GSC lines treated with GSK591 or SGC2096 are available on EGA under the accession ID EGAS00001004397 [https://www.ebi.ac.uk/ega/studies/EGAS00001004397]. The RT-PCR data with capillary electropherograms and PSI calculations can be accessed here: https://rnomics-store.med.usherbrooke.ca/palace//data/related/3289. The mass spectrometry proteomics data have been deposited to the ProteomeXchange Consortium via the PRIDE partner repository with the dataset identifier PXD021635 [http://www.ebi.ac.uk/pride/archive/projects/PXD021635]. The raw GSK591 and LLY-283 dose response data is available through https://github.com/bhklab/PRMT5i_GBM. Source data are provided with this paper.

Field-specific reporting

Please select the one below that is the best fit for your research. If you are not sure, read the appropriate sections before making your selection.

☒ Life sciences ☐ Behavioural & social sciences ☐ Ecological, evolutionary & environmental sciences

For a reference copy of the document with all sections, see nature.com/documents/nr-reporting-summary-flat.pdf

Life sciences study design

All studies must disclose on these points even when the disclosure is negative.

Sample size	No sample size calculations were performed. The sample size for each experiment is provided in the figure legends and in the main manuscript. Sample sizes were chosen to enable confident and meaningful conclusions from the data. Sample sizes of 3 biological replicates and two or three individual experiments were sufficient to determine significant differences for changes in the analyzed biological assays. For in vitro experiments with patient derived samples, we used a minimum of 3 independent patient-derived samples. However, for certain experiments where we saw high levels of variability, (for example for testing dose responses to drug), we tested as many samples as were practically feasible (46 patient-derived samples).
Data exclusions	One mouse was excluded from the in vivo survival curve calculation as it developed a tumor outside the skull. We believe this is due to technical error in injection.
Replication	All in vitro experiments were performed with a minimum of n=3 technical replicates in a minimum of 3 independent cell lines and a minimum of 3 independent experiments. Where patient samples are involved, experiments were performed with a minimum of 3 independent patient samples. All findings were reproducible over several experiments.
Randomization	Mice injected with patient-derived GBM stem cells were randomized into two groups on the basis of age and day of injection. Randomization was not relevant to the in vitro studies as each group consisted of treatment with distinct compounds/ vehicle.
Blinding	For Limiting Dilution Analysis using small molecules, the investigator was aware of the cell lines being used, but was blinded to what agents they were being treated with until after scoring. A similar approach was used for all other in vitro assays.

Reporting for specific materials, systems and methods

We require information from authors about some types of materials, experimental systems and methods used in many studies. Here, indicate whether each material, system or method listed is relevant to your study. If you are not sure if a list item applies to your research, read the appropriate section before selecting a response.

Materials & experimental systems

n/a	Involved in the study
☐	☒ Antibodies
☐	☒ Eukaryotic cell lines
☒	☐ Palaeontology
☐	☒ Animals and other organisms
☐	☒ Human research participants
☒	☐ Clinical data

Methods

n/a	Involved in the study
☒	☐ ChIP-seq
☒	☐ Flow cytometry
☒	☐ MRI-based neuroimaging

Antibodies

Antibodies used	Symmetric Di-Methyl Arginine Motif [anti-SDMA] MultiMab™ Rabbit mAb mix #13222, Cell Signaling Technologies, Cat#13222.<br>anti-GAPDH antibody: EMD Millipore, Cat #: MAB374, clone 6C5, anti MTAP antibody #4158, Cell Signaling Technologies, human p16INK4a/CDKN2A antibody AF5779, R&D Systems.<br>2ndary Antibodies: IRDye® 680RD anti-mouse IgG LI-COR Cat #926-68072<br>IRDye® 800CW anti-rabbit IgG LI-COR Cat #926-32211
Validation	anti-SDMA Ab validation: we run Western blots using cell lysates from human GBM patient-derived cells treated with 2 independent PRMT5 inhibitors and their inactive controls. The PRMT5 inhibitor treated cell lysates show significant reductions in the SDMA signal. Results are shown in figures 1e, 6d, supplementary Figures 1e and 2g. The SDMA signal shows dose-dependent decrease in intensity with increasing doses of GSK591 or LLY-283 (Supplementary Fig 1e). We also tested PRMT5 siRNA knockdowns in several cancer cell lines and the SDMA levels were greatly reduced. Moreover, this Ab has been widely cited in the literature: see PMIDs 32025719, 30916320, 30811983, 29706550, 31390828, 29227283, 27183006, 26258414, 31257072.<br>MTAP and p16INK4a antibodies: we validated them across a large panel of patient-derived GBM lines with known genotypes that had 2 copies or were homozygous deleted for the MTAP and CDKN2A loci (blots are shown in Figure 2c). Furthermore, each antibody has been widely used in the literature. Citations for the MTAP Ab: PMIDs 31257072, 31533041, 30916320, 31249865, 26909863. Citations (PMIDs) for p16INK4A/CDKN2A antibody: 31825831, 29525471, 28270683, 27349869, 28980705.<br>Anti-Glyceraldehyde-3-Phosphate Dehydrogenase Antibody, clone 6C5 is a well published and extensively characterized monoclonal antibody. This antibody has been verified using one of the 5 pillars of antibody validation (Uhlen et al., Nature, 2016). This purified mAb detects Glyceraldehyde-3-Phosphate Dehydrogenase (GAPDH) & has been published &

validated for use in ELISA, IP, IC, IF, IH & WB in multiple species. There are hundreds of citations with this Ab on the manufacturer's website. Select PMIDs include: 26230519, 25174395, 25230394, 26448624, 25505141, 25510912, 25723488, 25749033, 25932647, 26092128. For a complete list of citations see: https://www.emdmillipore.com/CA/en/product/Anti-Glyceraldehyde-3-Phosphate-Dehydrogenase-Antibody-clone-6C5,MM_NF-MAB374#anchor_REF

Eukaryotic cell lines

Policy information about cell lines

Cell line source(s)	The cells used were derived from surgical specimens from patients with Glioblastoma
Authentication	Authentication was done with PCR using a panel of polymorphic markers. The lines were matching the patient they were derived from.
Mycoplasma contamination	All cell lines were tested for mycoplasma contamination and they were confirmed to be mycoplasma-free.
Commonly misidentified lines (See ICLAC register)	*Name any commonly misidentified cell lines used in the study and provide a rationale for their use.*

Palaeontology

Specimen provenance	*Provide provenance information for specimens and describe permits that were obtained for the work (including the name of the issuing authority, the date of issue, and any identifying information).*
Specimen deposition	*Indicate where the specimens have been deposited to permit free access by other researchers.*
Dating methods	*If new dates are provided, describe how they were obtained (e.g. collection, storage, sample pretreatment and measurement), where they were obtained (i.e. lab name), the calibration program and the protocol for quality assurance OR state that no new dates are provided.*

☐ Tick this box to confirm that the raw and calibrated dates are available in the paper or in Supplementary Information.

Animals and other organisms

Policy information about studies involving animals; ARRIVE guidelines recommended for reporting animal research

Laboratory animals	4-20 weeks old female NSG mice were housed in an animal facility at a temperature 25 +/- 2 C, 45-55% humidity, and a light cycle of 6am on, 8pm off.
Wild animals	No wild animals were used in this study.
Field-collected samples	**No field collected samples were used in this study.**
Ethics oversight	**All animal protocols described in this study were approved by the Animal Care Committee at The Hospital for Sick Children.**

Note that full information on the approval of

Human research participants

Policy information about studies involving human research participants

Population characteristics	Glioblastoma tissues (and derived lines) were obtained from surgical specimens from patients recruited from St. Michael's Hospital (Toronto, ON, Canada), Toronto Western Hospital (Toronto, ON, Canada), The Hospital for Sick Children (Toronto, ON, Canada), and the University of Calgary (Calgary, AB, Canada), with informed consent. Study inclusion was not dependent on any other patient characteristics (i.e. age, gender, etc).
Recruitment	**Participants were recruited following informed consent**
Ethics oversight	**REB 0020010404 and REB 0020020238 were approved by The Arthur and Sonia Labatt Brain Tumour Research Centre at The Hospital for Sick Children (Toronto, ON, Canada) and REB HREBA-CC-160762 was approved at the University of Calgary (Calgary, AB, Canada).**

Note that full information on the approval of the study protocol must also be provided in the manuscript. *Identify the organization(s) that approved the s*

Clinical data

Policy information about clinical studies

All manuscripts should comply with the ICMJE guidelines for publication of clinical research and a completed CONSORT checklist must be included with all submissions.

Clinical trial registration	*Provide the trial registration number from ClinicalTrials.gov or an equivalent agency.*
Study protocol	*Note where the full trial protocol can be accessed OR if not available, explain why.*

Data collection	*Describe the settings and locales of data collection, noting the time periods of recruitment and data collection.*
Outcomes	*Describe how you pre-defined primary and secondary outcome measures and how you assessed these measures.*

ChIP-seq

Data deposition

☐ Confirm that both raw and final processed data have been deposited in a public database such as GEO.

☐ Confirm that you have deposited or provided access to graph files (e.g. BED files) for the called peaks.

Data access links<br>*May remain private before publication.*	*For "Initial submission" or "Revised version" documents, provide reviewer access links. For your "Final submission" document, provide a link to the deposited data.*
Files in database submission	*Provide a list of all files available in the database submission.*
Genome browser session<br>(e.g. UCSC)	*Provide a link to an anonymized genome browser session for "Initial submission" and "Revised version" documents only, to enable peer review. Write "no longer applicable" for "Final submission" documents.*

Methodology

Replicates	*Describe the experimental replicates, specifying number, type and replicate agreement.*
Sequencing depth	*Describe the sequencing depth for each experiment, providing the total number of reads, uniquely mapped reads, length of reads and whether they were paired- or single-end.*
Antibodies	*Describe the antibodies used for the ChIP-seq experiments; as applicable, provide supplier name, catalog number, clone name, and lot number.*
Peak calling parameters	*Specify the command line program and parameters used for read mapping and peak calling, including the ChIP, control and index files used.*
Data quality	*Describe the methods used to ensure data quality in full detail, including how many peaks are at FDR 5% and above 5-fold enrichment.*
Software	*Describe the software used to collect and analyze the ChIP-seq data. For custom code that has been deposited into a community repository, provide accession details.*

Flow Cytometry

Plots

Confirm that:

☐ The axis labels state the marker and fluorochrome used (e.g. CD4-FITC).

☐ The axis scales are clearly visible. Include numbers along axes only for bottom left plot of group (a 'group' is an analysis of identical markers).

☐ All plots are contour plots with outliers or pseudocolor plots.

☐ A numerical value for number of cells or percentage (with statistics) is provided.

Methodology

Sample preparation	*Describe the sample preparation, detailing the biological source of the cells and any tissue processing steps used.*
Instrument	*Identify the instrument used for data collection, specifying make and model number.*
Software	*Describe the software used to collect and analyze the flow cytometry data. For custom code that has been deposited into a community repository, provide accession details.*
Cell population abundance	*Describe the abundance of the relevant cell populations within post-sort fractions, providing details on the purity of the samples and how it was determined.*
Gating strategy	*Describe the gating strategy used for all relevant experiments, specifying the preliminary FSC/SSC gates of the starting cell population, indicating where boundaries between "positive" and "negative" staining cell populations are defined.*

☐ Tick this box to confirm that a figure exemplifying the gating strategy is provided in the Supplementary Information.

Magnetic resonance imaging

Experimental design

Design type	*Indicate task or resting state; event-related or block design.*
Design specifications	*Specify the number of blocks, trials or experimental units per session and/or subject, and specify the length of each trial or block (if trials are blocked) and interval between trials.*
Behavioral performance measures	*State number and/or type of variables recorded (e.g. correct button press, response time) and what statistics were used to establish that the subjects were performing the task as expected (e.g. mean, range, and/or standard deviation across subjects).*

Acquisition

Imaging type(s)	*Specify: functional, structural, diffusion, perfusion.*
Field strength	*Specify in Tesla*
Sequence & imaging parameters	*Specify the pulse sequence type (gradient echo, spin echo, etc.), imaging type (EPI, spiral, etc.), field of view, matrix size, slice thickness, orientation and TE/TR/flip angle.*
Area of acquisition	*State whether a whole brain scan was used OR define the area of acquisition, describing how the region was determined.*

Diffusion MRI ☐ Used ☐ Not used

Preprocessing

Preprocessing software	*Provide detail on software version and revision number and on specific parameters (model/functions, brain extraction, segmentation, smoothing kernel size, etc.).*
Normalization	*If data were normalized/standardized, describe the approach(es): specify linear or non-linear and define image types used for transformation OR indicate that data were not normalized and explain rationale for lack of normalization.*
Normalization template	*Describe the template used for normalization/transformation, specifying subject space or group standardized space (e.g. original Talairach, MNI305, ICBM152) OR indicate that the data were not normalized.*
Noise and artifact removal	*Describe your procedure(s) for artifact and structured noise removal, specifying motion parameters, tissue signals and physiological signals (heart rate, respiration).*
Volume censoring	*Define your software and/or method and criteria for volume censoring, and state the extent of such censoring.*

Statistical modeling & inference

Model type and settings	*Specify type (mass univariate, multivariate, RSA, predictive, etc.) and describe essential details of the model at the first and second levels (e.g. fixed, random or mixed effects; drift or auto-correlation).*
Effect(s) tested	*Define precise effect in terms of the task or stimulus conditions instead of psychological concepts and indicate whether ANOVA or factorial designs were used.*

Specify type of analysis: ☐ Whole brain ☐ ROI-based ☐ Both

Statistic type for inference (See Eklund et al. 2016)	*Specify voxel-wise or cluster-wise and report all relevant parameters for cluster-wise methods.*
Correction	*Describe the type of correction and how it is obtained for multiple comparisons (e.g. FWE, FDR, permutation or Monte Carlo).*

Models & analysis

n/a	Involved in the study
☐	☐ Functional and/or effective connectivity
☐	☐ Graph analysis
☐	☐ Multivariate modeling or predictive analysis

Functional and/or effective connectivity	*Report the measures of dependence used and the model details (e.g. Pearson correlation, partial correlation, mutual information).*
Graph analysis	*Report the dependent variable and connectivity measure, specifying weighted graph or binarized graph, subject- or group-level, and the global and/or node summaries used (e.g. clustering coefficient, efficiency, etc.).*

Multivariate modeling and predictive analysis

Specify independent variables, features extraction and dimension reduction, model, training and evaluation metrics.
